# Synergistic Effects of Boron Nitride (BN) Nanosheets and Silver (Ag) Nanoparticles on Thermal Conductivity and Electrical Properties of Epoxy Nanocomposites

**DOI:** 10.3390/polym12020426

**Published:** 2020-02-12

**Authors:** Yunjian Wu, Xiaoxing Zhang, Ankit Negi, Jixiong He, Guoxiong Hu, Shuangshuang Tian, Jun Liu

**Affiliations:** 1 School of Electrical Engineering and Automation, Wuhan University, Wuhan 430072, China; wuyunjian@whu.edu.cn (Y.W.); 15733221344@163.com (G.H.); 2 Department of Mechanical and Aerospace Engineering, North Carolina State University, Raleigh, NC 27695, USA; anegi2@ncsu.edu (A.N.); jhe17@ncsu.edu (J.H.); 3 School of Electrical and Electronic Engineering, Hubei Key Laboratory for High-efficiently Utilization of Solar Energy and Operation Control of Energy Storage System, Hubei University of Technology, Wuhan 430068, China; tianshuang1002@yahoo.com

**Keywords:** polymer nanocomposites, thermal conductivity, electrical properties, silver nanoparticles/boron nitride nanosheets, PACS:

## Abstract

Polymer composites, with both high thermal conductivity and high electrical insulation strength, are desirable for power equipment and electronic devices, to sustain increasingly high power density and heat flux. However, conventional methods to synthesize polymer composites with high thermal conductivity often degrade their insulation strength, or cause a significant increase in dielectric properties. In this work, we demonstrate epoxy nanocomposites embedded with silver nanoparticles (AgNPs), and modified boron nitride nanosheets (BNNSs), which have high thermal conductivity, high insulation strength, low permittivity, and low dielectric loss. Compared with neat epoxy, the composite with 25 vol% of binary nanofillers has a significant enhancement (~10x) in thermal conductivity, which is twice of that filled with BNNSs only (~5x), owing to the continuous heat transfer path among BNNSs enabled by AgNPs. An increase in the breakdown voltage is observed, which is attributed to BNNSs-restricted formation of AgNPs conducting channels that result in a lengthening of the breakdown path. Moreover, the effects of nanofillers on dielectric properties, and thermal simulated current of nanocomposites, are discussed.

## 1. Introduction

Miniaturization of power equipment and electronic devices, coupled with a high voltage electromagnetic environment, demands superior thermal conductivity and electrical insulation strength in insulating materials [1,2,3]. An ideal insulating material is expected to possess high thermal conductivity, high breakdown strength, low permittivity, and low dielectric loss, simultaneously [4]. Polymers are widely used as insulating materials for their excellent electrical insulation properties [5,6], but their low thermal conductivity (~0.2 W m^−1^ K^−1^ at room temperature) reduces the lifetime and reliability of power equipment and electronic devices [7,8,9].

To improve the thermal conductivity of polymers, many kinds of nanomaterials with high thermal conductivity have been added as fillers in the literature [10,11,12]. However, obtaining significant improvement in thermal conductivity without undermining insulation properties is challenging [13]. Nanofillers, such as carbon nanotubes [14], graphene [15], and metals (such as copper [16]) leads to the decrease of insulation strength. Some ceramic fillers, including aluminum oxide [17], aluminum nitride [18], silicon carbide [19], and strontium titanate [20] induce a sharp rise in dielectric properties, while others, such as silicon dioxide, limit thermal conductivity enhancement [21].

Boron nitride nanosheets (BNNSs) are promising for thermally conductive insulating materials, not only because they have intrinsically high thermal conductivity, but because they have a wide band gap of 5.9 eV, with excellent electrical insulating properties, such as high breakdown strength, low electrical conductivity, and low dielectric parameters [22,23]. However, BNNSs are difficult to form a thermal transfer network in polymer matrix alone. If the BNNSs were connected by nanoparticles to form an inter-filler thermal network in polymer composites, the thermal conductivity would be effectively improved at relatively low content [24,25,26]. Silver nanoparticles (AgNPs) are potentially used as the connecting nanoparticles, owing to their outstanding thermal conductivity and simpler distribution on the surface of BNNSs by silver nitrate reduction [27,28]. Furthermore, the AgNPs on the surface of BNNSs can be electrically blocked by BNNSs, effectively [29,30].

In this work, we chose epoxy as the polymer matrix that is widely used in insulating material, and BNNSs and AgNPs as the nanofillers. By placing AgNPs on the surface of BNNSs, and then adding the hybrid nanofiller into the epoxy matrix, we successfully synthesized an epoxy nanocomposite (EP-AgBN) with superb thermal conductivity, high insulation strength, low permittivity, and low dielectric loss. The synergistic effects of BNNSs and AgNPs in epoxy nanocomposites on thermal conductivity and electrical properties are studied. It is expected that the EP-AgBN exhibits strong potential as an insulating material for power equipment and electronic devices, such as insulators, flexible substrates, and electronic packages.

## 2. Experimental Section

### 2.1. Materials

Hexagonal boron nitride (h-BN) (size of 1~2 μm) was purchased from Aladdin, China. N, N-dimethylformamide (DMF, purity ≥99.5%), silver nitrate (purity ≥99.8%), alcohol (purity ≥99.9%), and acetone (purity ≥99.95%) were purchased from Sinopharm Chemical Reagent, China. E-51 epoxy resin (2,2′-[(1-methylethylidene)bis(4,1-phenyleneoxymethylene)]bis-oxiranhomopol) and 593 curing agent (adduct of diethylenetriamine and butyl glycidyl ether) were purchased from Shengshi Chemical, wuhan, China. All materials were used as received.

### 2.2. Preparation of AgNPs-BNNSs

Liquid-phase exfoliation of h-BN was used to prepare BNNSs. In the exfoliation process, 1.5 g h-BN was dissolved in a sufficient amount of DMF (300 mL), then the mixture was under continuous sonication for 72 h. During this period, h-BN was gradually exfoliated into BNNSs. Seventy-two hours later, a solution of 1.5 g silver nitrate and 15 mL DMF was dropped slowly into the exfoliation solution that was stirred and sonicated synchronously. This process would last for 2 h, and the mixture was kept without any operation, in room temperature for 24 h, to ensure the uniform distribution of AgNPs on the surface of BNNSs. Finally, the solution was filtered, and repeatedly washed with alcohol and acetone to obtain the AgNPs-BNNSs. In this method, the DMF acts as both the exfoliation liquid and reducing agent.

### 2.3. Preparation of EP-AgBN

The AgNPs-BNNSs was added into acetone (1:15 mass ratio) and sonication was used to mix them uniformly. After the addition of E-51 epoxy (20 g), the mixture was stirred continuously under 70 °C for 7 h to volatilize all the acetone. Next, the mixture was subjected to sonication, and stirring simultaneously with the addition of the 593 curing agent at 30 °C for 2 h, the mass ratio of epoxy and curing agent was 4:1. Finally, the mixture was degassed in a vacuum box for 1 h. The curing process was in a mold at 100 °C under high pressure for 5 h to obtain composites of EP-AgBN. By controlling the content of AgNPs-BNNSs, we obtained composites with different nanofiller content of 5, 10, 15, 20, and 25 vol%, respectively.

### 2.4. Morphology Characterization And Performances Measurement

X-ray diffraction (XRD) was used to determine the elements, impurity, and crystal planes, with the scanning angle and scanning speed of X-ray diffractometer (Xpert Pro, Netherlands) as 2θ = 10°–80° and 0.02°/s, respectively.

The morphology of AgNPs-BNNSs was taken by transmission electron microscopy (TEM) (JEM-2100, Tokyo, Japan) and the acceleration voltage was 200 kV.

The morphology of EP-AgBN was obtained by scanning electron microscope (SEM) (QUANTA, Eindhoven, the Netherlands), and the accelerating voltage was 30 kV.

The X-ray photoelectron spectroscopy (XPS) was obtained by X-ray photoelectron spectrometer (ESCALAB250Xi, Waltham, Massachusetts, USA), and the applied voltage was 15 kV.

The thermal conductivity was measured by a fully automatic thermal conductivity meter (DRL-III, Xiangtan, China), which uses the steady-state heat flow method, according to the ASTM D5470-2006 standard.

The dielectric properties were measured by a broadband dielectric spectrometer (BDS) (Concept 80, Montabaur, Germany) at room temperature from 1 Hz to 1 MHz.

The electric breakdown voltage of all samples was tested using the plate electrode, with a radius 1 cm, and the frequency of the AC was 50Hz. Eight samples were tested repeatedly for each composite.

The thermal simulated current (TSC) was measured by a thermal stimulated current analyzer (SETARAM Ⅱ, Lyon, France). Firstly, the temperature was heated to the polarization temperature of 40 °C. Next, the polarization voltage of 300 V was applied and kept for 20 min. After that, the temperature was rapidly decreased to −100 °C at a rate of 30 °C/min. The polarization voltage was removed at −100 °C and kept for 10 min. Finally, temperature slowly increased to 120 °C at a rate of 2 °C/min, and the current recorded in this step was the TSC of the sample.

## 3. Results and Discussion

### 3.1. Morphology Characterization of AgNPs-BNNSs

**Figure 1:**
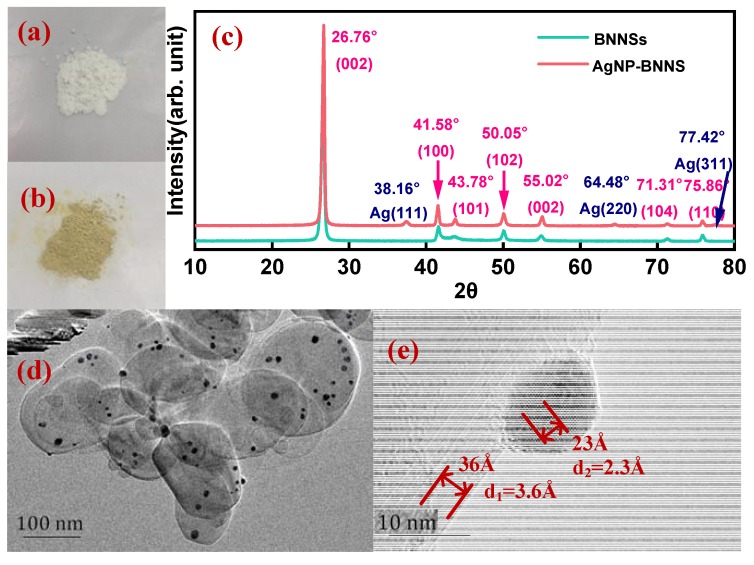
Optical images of (**a**) hexagonal boron nitride (h-BN) and (**b**) silver nanoparticles-boron nitride nanosheets (AgNPs-BNNSs); (**c**) XRD spectrum of BNNSs and AgNPs-BNNSs; (**d**) morphology of AgNP-BNNS by TEM; (**e**) The TEM atomic image of BNNSs and AgNPs with lattice spacing labeled.

Figure 1a,b show the optical images of h-BN and AgNPs-BNNSs. The h-BN is white and the prepared AgNPs-BNNSs is yellow. Figure 1c shows the XRD patterns of BNNS by liquid-phase exfoliation and AgNPs-BNNSs. There are no impurity peaks in the spectrum, which indicates that the nanomaterials are highly purified. There exists diffraction peaks at 2θ ≈ 26.76°, 41.58°, 43.78°, 50.05°, 55.02°, 71.31°, and 75.86° for both BNNSs and AgNPs-BNNSs, which correspond to the crystal planes (002), (100), (101), (102), (004), (104), and (110) of h-BN, respectively [30,31]. Also, there are three diffraction peaks at 2θ ≈ 38.16°, 64.48°, and 77.42° of AgNPs-BNNSs, which are the planes (111), (220), and (311) of silver crystals [32,33], which prove that the prepared AgNPs-BNNSs contain silver nanoparticles. Figure 1d,e show the morphology of AgNPs-BNNSs. In Figure 1d, h-BN is exfoliated to regular sheet-like BNNSs and the black spots distributed on the surface of BNNSs are AgNPs. Figure 1e shows the atomic image of the composites by TEM. The lattice spacing of the sheet is 3.6 Å and the black spots are 2.3 Å, corresponding to the crystal plane (002) of BNNSs and the crystal plane (111) of AgNPs, respectively. All of these features further prove that the prepared composite products are AgNPs modified BNNSs.

### 3.2. Morphology Characterization of EP-AgBN

**Figure 2:**
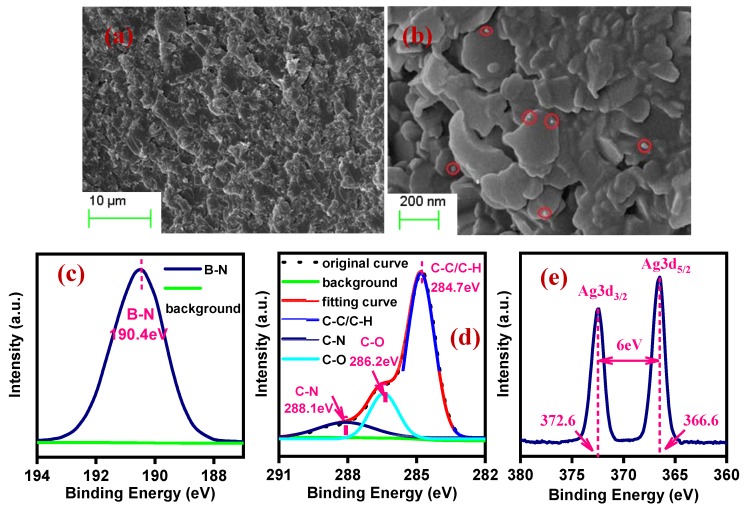
(**a**,**b**) Morphology of epoxy (EP)-AgBN with a nanofiller content of 25 vol% by SEM with different scales; XPS spectra of EP-AgBN; (**c**) narrow B 1s scans of EP-AgBN; (**d**) narrow C 1s scans of EP-AgBN; (**e**) narrow Ag 3d scans of EP-AgBN.

Figure 2a shows the fracture surface image of epoxy composite with a nanofiller content of 25 vol% by SEM. Owing to the vacuum degassing and high-pressure curing, the nanofillers are homogeneously dispersed and maintain strong adhesion with the epoxy matrix. Figure 2b shows that the nanoparticles are attached to the BNNSs surface. Several nanoparticles are observed with relatively larger size in comparison to Figure 1d, as small particles combine into larger ones to reduce surface energy during composite preparation. The XPS spectrum can reveal the composition of elements contained in a substance surface accurately. Figure 2c,d,e show the XPS spectrum of EP-AgBN. The peak at 190.4 eV represents the B–N bond and the absorption curves of C–C/C–H (284.7 eV), C–O (286.2 eV) and C–N (288.1 eV) are obtained by peak separation at 284.7 eV, which are the chemical bonds in the epoxy matrix [34,35]. Moreover, energy bandwidth between Ag3d5/2 (366.6 eV) and Ag3d3/2 (372.6 eV) is 6 eV, meaning that the AgNPs are distributed on the surface of BNNSs in a form of granular [34].

### 3.3. Thermal Conductivity of EP-AgBN

**Figure 3:**
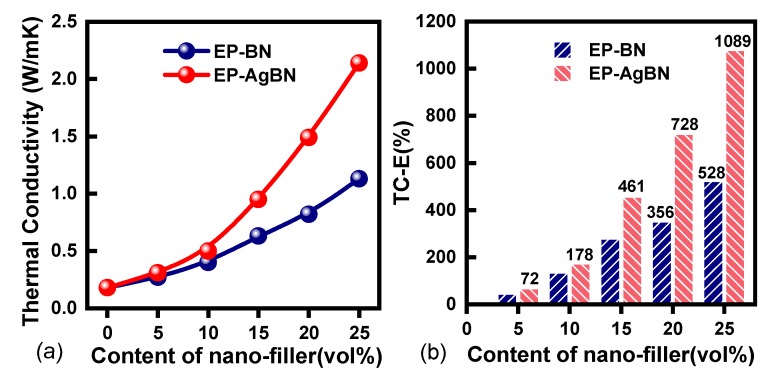
(**a**) Thermal conductivity of EP-BN and EP-AgBN; (**b**) Thermal conductivity enhancement of EP-BN and EP-AgBN.

Figure 3a shows the effect of nanofiller content on thermal conductivity for EP-BN and EP-AgBN. The measured thermal conductivity of neat epoxy is 0.18 W m^−1^ K^−1^. Improvement in thermal conductivity is realized for both the nanofiller composites. The magnitude of rise is similar in both cases for low nanofiller content (<10 vol%). This is primarily due to insufficient amount of AgNPs to significantly influence thermal conductivity. Beyond 10 vol% of nanofillers, however, EP-AgBN shows great thermal conductivity improvement compared to EP-BN. Moreover, the thermal conductivity of EP-AgBN reaches to 2.14 W m^−1^ K^−1^ with a filler loading of 25 vol%, which is nearly twice to that of EP-BN (1.13 W m^−1^ K^−1^). This indicates that AgNPs-BNNSs are superior to BNNSs for improving the thermal conductivity of epoxy.

Figure 3b shows the thermal conductivity enhancement (TC-E) for EP-AgBN and EP-BN according to the formula TC-E=(Ki-Kneat_epoxy/Kneat_epoxy, where Ki is the thermal conductivity of composite and Kneat_epoxy is the TC of pure epoxy. Limited TC-E is achieved in both composites for low nanofiller loading (<10 vol%). Noticeable difference in TC-E between the composites is observed at higher nanofiller contents.

**Figure 4:**
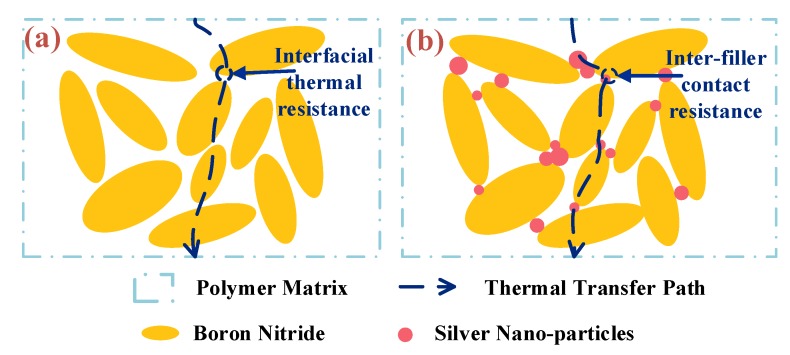
Thermally conductive path of polymer nanocomposites (**a**) without inter-filler neatwork; (**b**) with inter-filler network.

Figure 4a,b illustrate the synergistic effect of BNNSs and AgNPs on thermal conductivity of nanocomposites. The BNNSs formed the main thermal transfer paths in nanocomposites and AgNPs connect the BNNS as a “thermal bridge”, which builds the inter-filler thermal network in polymer composites. Nanocomposites with inter-filler network possess a higher thermal conductivity than those without a network as the inter-filler contact resistance is much less than the interfacial thermal resistance between matrix and filler.

In Table 1, we summarized the thermal conductivity of nanocomposites with different BN-based fillers. Compared with others, the thermal conductivity of EP-AgBN is preferable.

### 3.4. Electrical Conductivity and Breakdown Voltage of EP-AgBN

**Figure 5:**
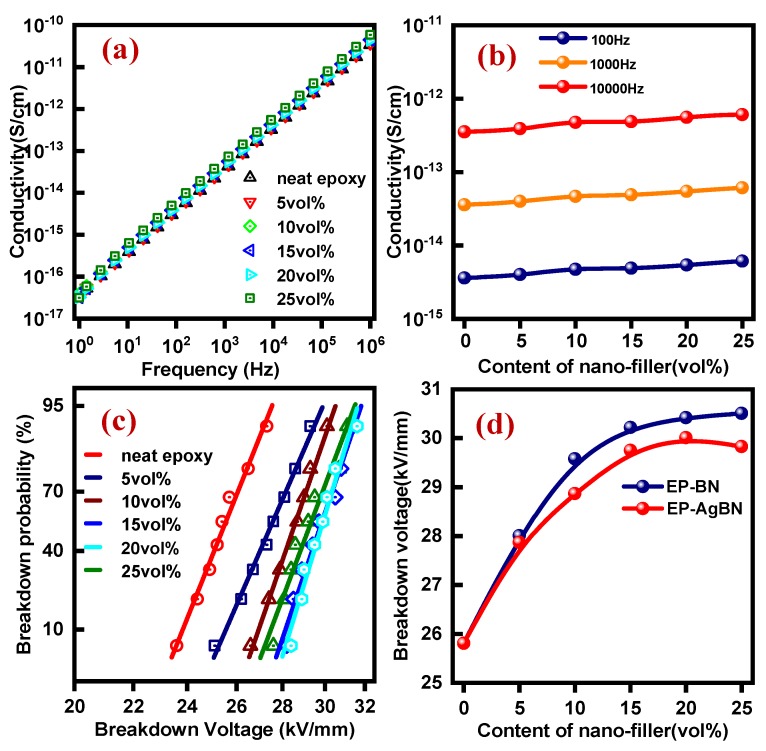
(**a**) Conductivity of EP and EP-AgBN at room temperature; (**b**) conductivity under 100 Hz, 1000 Hz, and 10,000 Hz; (**c**) Weibull distribution of the breakdown voltage; (**d**) breakdown voltage of EP-BN and EP-AgBN.

Insulation materials are expected to possess low electrical conductivity and high breakdown voltage in engineering applications. Figure 5a shows the linear electrical conductivity behavior for different composites (10^−10^–10^−17^ Scm^−1^) over a broad frequency range. The electrical conductivity is within the desired range for insulating materials (10^−9^–10^-20^ Scm^−1^). Figure 5b shows the electrical conductivity of EP-AgBN for three different frequencies: low frequency (100 Hz), intermediate frequency (1000 Hz), and high frequency (10,000 Hz). As expected, the electrical conductivity increases with the addition of conducting nanofillers; however, this increase is limited due to the presence of highly insulating BNNSs, which restricts the aggregation of AgNPs.

Figure 5c shows the Weibull distribution of the breakdown voltage. The breakdown voltage corresponding to a breakdown probability of 63.2% is taken as the breakdown voltage of the composite, as shown in Figure 5d. For EP-BN, the breakdown voltage grows at a higher rate initially from 25.81 kV mm^−1^ and nearly saturates at around 30.51 kV mm^−1^ at 25 vol% nanofiller. In the case of EP-AgBN, the breakdown voltage achieves a maximum value, and then decays little due to electricity conductivity of silver nanoparticles. The breakdown voltage magnitude is still 4.02 kV mm^−1^ higher as compared to neat epoxy. The breakdown voltage of EP-BN is marginally higher than that of EP- AgBN for the same nanofiller content due to the presence of silver nanoparticles. Compared to neat epoxy, the EP-AgBN has a superior insulation strength for the nanofiller content of 25 vol%.

**Figure 6:**
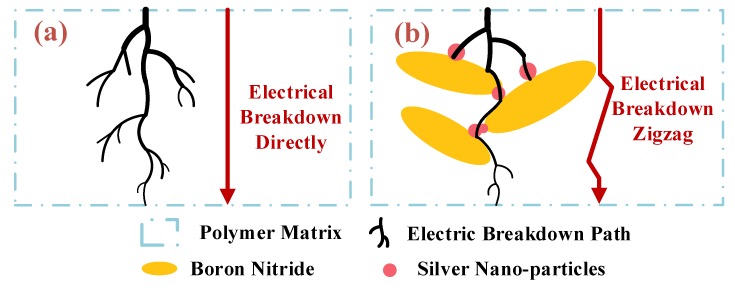
Electric breakdown path of (**a**) neat polymer; (**b**) polymer nanocomposites.

Figure 6a and b illustrates the synergistic effect of BNNSs and AgNPs on electric breakdown of nanocomposites. In Figure 6a, the polymer will directly breakdown without nanofillers. In Figure 6b, the AgNPs are blocked by BNNSs, which makes it difficult to form an electrically conductive channel. As a result, the breakdown path is forced into a “zig-zag” shape in EP-AgBN and the breakdown distance is elongated, thereby intensifying the insulation strength.

### 3.5. Permittivity and Dielectric loss of EP-AgBN

**Figure 7:**
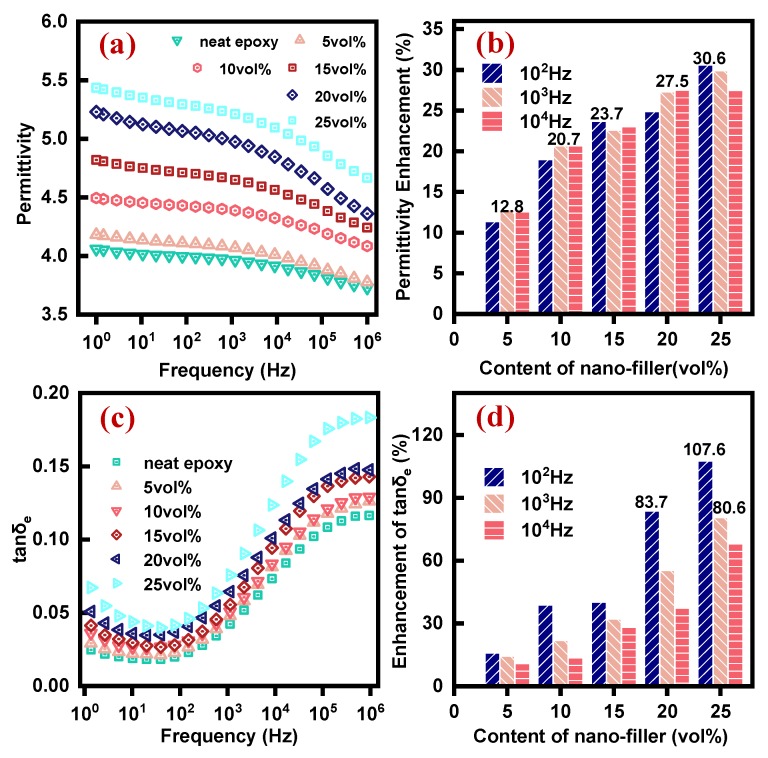
(**a**) Permittivity of EP and EP-AgBN at room temperature; (**b**) permittivity enhancement of EP-AgBN under 100, 1000, and 10,000 Hz; (**c**) tangent of dielectric loss angle of EP and EP-AgBN; (**d**) enhancement of tanδe of EP-AgBN under 100, 1000, and 10,000 Hz.

Improvement in thermal conductivity and insulation strength is often accompanied by undesirable dielectric properties [41]. Low permittivity and dielectric loss are typical requirements for electrically insulating materials. Figure 7a shows low permittivity (~3.6–5.5) in EP-AgBN over a broad frequency range. For pure epoxy, the polarization is dipole polarization under the electric field. The pure epoxy creates a 3D network structure which prevents polarization in certain polar groups, thus leading to low permittivity [42]. Addition of AgNP-BNNSs triggers an increase in permittivity due to the presence of interfacial polarization and electron polarization [12,43,44]. The decrease of permittivity with frequency is also obtained. This can be explained by the decrease of dipole polarization and interface polarization because they both have a small relaxation frequency and cannot keep up with the change of frequency at the high frequency [12].

Figure 7b shows the permittivity enhancement under three different frequencies. Enhancement in permittivity is fairly low with a maximum value of 30.6%. This behavior is expected due to the low permittivity of BNNS itself. In addition to that, composites prepared in this work are uniform with strong nanofillers-matrix bonding which only causes mild enhancement in the interfacial polarization strength.

Figure 7c shows the room temperature dielectric loss angle (tanδe) for EP-AgBN as a function of frequency. Low dielectric loss (<0.2) for composites indicates that superior dielectric properties of neat epoxy are still preserved. With the addition of nanofillers, the dielectric loss of EP-AgBN grows due to the increase of interfacial polarization, electron polarization loss, and loss of the conduction current formation. The spectral variation of tanδe is bifurcated into low (1–100 Hz) and high frequency (>100 Hz) bands owing to significantly different behaviors. Reduction in the dipole polarization and interface polarization explains the slightly decreasing tanδe trend in the former band [41]. The rapid rise in the high frequency region is assigned to the orientation movement acceleration of dipole, and increase of heat generation by friction between the epoxy segments [45].

Figure 7d shows increasing tanδe enhancement with nanofiller content for three different frequencies. For low nanofiller content, the tanδe enhancement is relatively small, as the majority of space charges are localized in deep traps of nanoparticles, making them difficult to separate, thus limiting charge transfer and leakage current [46]. Successive addition of nanofillers causes significant growth in tanδe due to enhanced charge transfer, leading to more leakage current. Furthermore, a gap between nanofiller and matrix also augments dielectric loss [40].

### 3.6. Thermal Simulated Current of EP-AgBN

**Figure 8:**
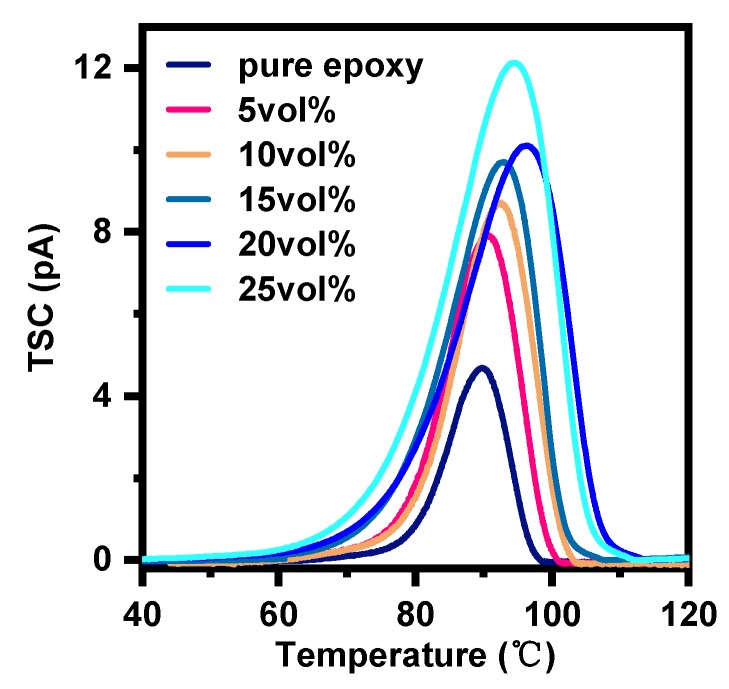
Thermal simulated current of pure epoxy and EP-AB.

TSC is a pivotal means to obtain the trap parameters and polarization properties of solid materials. Trap depth represents the ability to bound charge, and the polarization charges can reflect the degree of polarization [47,48]. Figure 8 shows the TSC of pure epoxy and EP-AgBN. Table 2 shows the calculated trap parameters according to the semi-peak method [49].

An increase is observed in the temperature of peak value form 4.76 pA of pure epoxy to 12.16 pA at a filler loading of 25 vol%. However, the temperature corresponding to peak value of TSC grows first with the content of Ag-BN below 20 vol% and then decreases slightly. The pure epoxy has a large trap depth of 3.285 eV, meaning that it possesses a strong capacity to bind charges. The addition of BNNSs-AgNPs leads to a decrease in trap depth of EP-AgBN, indicating that the ability to bind charges is weakened, which coincides with the increase in permittivity. The trap depth gets a minimum value of 1.966 eV that belongs to deep trap (shallow trap is less than 1 eV), this can be used to explain why there is no sharp rise in dielectric properties of EP-AgBN. Moreover, the nanofillers fabricated in this paper intensifies the polarization of EP-AgBN, resulting in an increase of the polarization charges, which coincides with the increase in dielectric loss.

## 4. Conclusions

To sum up, we successfully achieved nanocomposites with high thermal conductivity, high insulation strength, low permittivity, and low dielectric simultaneously in EP-AgBN, solving the problem that polymer composites with high thermal conductivity always have low insulation strength or high dielectric parameters. The intrinsic excellent insulating properties of BNNSs and the well-designed surface modification by AgNPs are proposed to be the key factors for obtaining outstanding insulation strength. Moreover, the nanofillers with electrical conductivity do not reduce the insulation properties of composites if they are effectively blocked by electrically insulating nanofillers. A nanofiller with better thermal conductivity can be obtained by placing nanofillers (such as graphene) with a small particle size on the surface of BNNSs. The excellent thermal and insulation properties of the present nanocomposites may pave the way for power equipment and electronic devices under a high energy density and complex electro-thermal operation environment, such as insulators, electronic packages, and solar power generation devices.

## Figures and Tables

**Table 1:** Thermal conductivity of composites with different BN-based fillers.

Matrix	Fillers	TC (m^-1^ K^-1^)	TC-E	Reference
epoxy	60wt% micro BN	1.052	420%	[3]
polypropylene	30vol% micro BN	~2	~900%	[12]
epoxy	25wt% BNNSs	0.65	180%	[36]
epoxy	25vol% BNNSs	1.13	528%	this paper
epoxy	30wt% h-BN	1.178	514%	[37]
polymethylmethacrylate	10wt% BNNTs	0.5	194	[38]
epoxy	22.5vol% micro+nano BN	1.4	250%	[39]
epoxy	25wt%BN +7.5wt%Al_2_O_3_	1.182	700%	[40]
epoxy	25.1vol%BNNS/AgNPs	3.05	1123%	[29]
epoxy	25vol% AgNPs-BNNSs	2.14	1089%	this paper

**Table 2:** Calculated trap parameters.

Sample	Peak Value (pA)	Temperature of Peak Value (°C)	Trap Depth(eV)	Polarization Charges (pC)
Pure epoxy	4.76	89.77	3.285	329.34
5vol%	8.04	90.61	2.770	663.24
10vol%	8.85	92.43	2.664	766.02
15vol%	9.73	92.96	2.353	956.04
20vol%	10.13	96.34	1.980	1205.28
25vol%	12.16	94.62	1.966	1441.92
